# Use of condensed molasses fermentation solubles as an alternative source of concentrates in dairy cows

**DOI:** 10.5713/ajas.19.0844

**Published:** 2020-02-25

**Authors:** Jian Ma, Chen Ma, Xue Fan, Ali Mujtaba Shah, Jiang Mao

**Affiliations:** 1 College of Animal Science, Xinjiang Agricultural University, Urumchi 100193, China; 2 Animal Nutrition Institute, Sichuan Agricultural University, Chengdu 611130, China; 3 Department of Livestock Production, Shaheed Benazir Bhutto University of Veterinary and Animal Sciences, Sakrand 67210, Pakistan; 4 New Hope Dairy Farming Co. LTD., Chengdu 610063, China

**Keywords:** Condensed Molasses Fermentation Solubles, Lactation Performance, Rumen Fermentation, Nutrient Digestibility, Lactating Cow

## Abstract

**Objective:**

The purpose of present study was to investigate the effects of condensed molasses fermentation solubles (CMS) on lactation performance, rumen fermentation, nutrient digestibility, and serum parameters of dairy cows.

**Methods:**

A total of 75 healthy Holstein cows with the same parity (milk production = 35±2.5 kg, body weight = 570±28 kg) were randomly selected and divided into 5 groups. One group served as control group (CON; no CMS), whereas the other 4 groups were CMS1 (accounted for 1% of the diet), CMS2 (2%), CMS3 (3%), and CMS4 (4%). All cows were fed regularly three times each day at 0800, 1600, and 2400 h. Cows received diet and water *ad libitum*. The experiment lasted for 60 days.

**Results:**

Results showed that the dry matter intake, milk yield, and protein of CMS2 were maximum and higher (p<0.05) than CMS4. The ruminal pH was observed less than 6 in CMS3 and CMS4 groups. No noticeable difference of microbial protein was found between CON and CMS2 groups, while the microbial protein in these groups was higher (p<0.05) than CMS3 and CMS4 groups. The apparent digestibility of dry matter, organic matter, and crude protein in CMS2 group was higher (p<0.05) than CMS3 and CMS4 groups. Compared to CMS3 and CMS4 groups, the CMS2 group increased (p<0.05) the serum concentrations of immunoglobulin G and immunoglobulin M on d 60.

**Conclusion:**

Therefore, it is practicable that CMS substitutes for a part of concentrates in lactating cows’ diets, but higher addition of CMS (more than 3% of the diet) could decrease production performance of dairy cows as seen in the present study.

## INTRODUCTION

Recently, there has been increasing attention given to by-products of the food industry resulting in their use as them as alternative feeds for ruminants due to enhanced environmental concerns and higher feed cost concerns [1,2]. Feeding industrial by-products to animals reduces the environmental impact of the food industry; besides, it improves the profitability of the industrial by-products [3]. Some by-products can be processed with fermentation to improve the quality, which increases their positive effects on animals [4]. In China, with the increase of feed cost, particularly for soybean meal, there is an irresistible trend to make full use of by-products, and the application of unconventional feed resources with high crude protein (CP) concentration and yield as alternative feeds of dairy cows is attracting increasing attention.

Molasses, commonly used as a feed additive, can improve the production performance [5] and rumen health [6] of dairy cows. Condensed molasses fermentation solubles (CMS) is made by microbial fermentation with sugarcane molasses as raw material. CMS is also rich in protein, amino acids, organic acids, vitamins, minerals, biochemical fulvic acid, and unknown growth factors synthesized by microorganisms in the fermentation process. Thus, compared to common molasses, CMS is an effective feed resource with high nutritional value and economic benefits. Previous studies have found that CMS could improve production performance [7] with economic benefits [8] for animals. However, some researchers have suggested that excessive CMS in diets could have adverse effects on animals’ health [9]. Therefore, it is important to evaluate the appropriate proportion of CMS in the diet.

Generally, the cost of concentrates occupies a higher percentage of feed cost. In the production of dairy cows, distillers dried grains with soluble (DDGS) can be used as a protein and energy feed for maintaining production performance of milking cows [10]. Beet pulp, a by-product of sugar manufacture, is a conventional energy feed in cows’ diet [11]. Furthermore, as an important protein feed, soybean meal is commonly used in the diet of dairy cows, especially for lactating cows. In China, soybean resources are scarce and need to be imported in large quantities every year. Thus, it is a burning issue to find resources that can replace concentrates, particularly soybean meal, in the diet of cows in modern large-scale intensive dairy farming. CMS is one such resources with a higher content of CP, and it is possible to use CMS to replace part of the concentrates in the diet of dairy cows. However, information on CMS replacing concentrates is scarce in commercial farms. Therefore, we hypothesized that the addition of CMS in a diet to partially replace concentrates could maintain lactation performance of milking cows. In addition, because of the negative effects of excessive CMS on animals, another objective of our work was to investigate the appropriate proportion of CMS in the diet of lactating cows.

## MATERIALS AND METHODS

### Animal ethics statement

The animal experiment was performed according to the Regulation on the Administration of Laboratory Animals (2017 Revision) promulgated by Decree No. 676 of the State Council. All procedures involving animal care and management were in accordance with and approved by the Institutional Animal Care and Use Committee of Xinjiang Agricultural University (Urumchi, Xinjiang, China).

### Experimental design and diets

The present experiment was conducted at a commercial dairy farm, which has approximately 4,000 milking cows. A total of 75 Holstein dairy cows with similar milk production (35± 2.5 kg), body weight (570±28 kg), days in milk (55±5 d), and same parity (second) were used in this study. The selected cows were randomly allocated into 5 dietary treatment groups: CON (no CMS [produced by Hongyuan Biological Fertilizer Co. Ltd., Yantai, Shandong, China], main nutrient contents: dry matter [DM], 58.6%; carbohydrates, 8.35%; CP, 30.1%; ether extract [EE], 0.95%), and CMS1 (accounted for 1% of the basal diet), CMS2 (2%), CMS3 (3%), and CMS4 (4%). The detailed substitute proportions of CMS for concentrates were as follows: for CMS1, replacement of CMS was 0.4% unit of soybean meal, 0.2% unit of DDGS, and 0.4% unit of beet pulp; for CMS2, CMS3, and CMS4, these substitute proportions were double, triple, and quadruple, respectively.

All the cows were fed regularly three times each day at 0800, 1600, and 2400 h with a total mixed ration (TMR). Cows had access to diet and water *ad libitum*. All cows were mechanically (Afimilk system) milked daily three times at 0600, 1400, and 2200 h. A 15-day transitional period followed by 60 days of formal experiment was implemented. CMS was supplemented in the concentrates and mixed with other ingredients. The basal diet was formulated according to NRC [12], and the ingredients and nutrient contents of basal diet are shown in Table 1.

### Sample collection

The amount of feed offered, and orts were recorded every day to determine the dry matter intake (DMI). DMI of every group was used to calculate the average daily DMI of per cow. Beginning at 0200 h on d 58, fecal samples were collected for 3 days (about 300 g) by stimulating the rectum to cause defecation. The sampling time was moved forward 2 h daily so that a sample was collected for each 2 h interval of one day. The specific time was as follows (d 58: 0200, 0800, 1400, and 2000 h; d 59: 0000, 0600, 1200, and 1800 h; d 60: 2200, 0400, 1000, and 1600 h) [13]. Meanwhile, feed and orts were sampled daily. The daily fecal samples, feed, and orts were mixed by per cow, subsampled, and then stored at −20°C until analysis. At the end of the experiment, all the samples were thawed (the 100 g fecal samples were mixed with 10 mL of 10% sulphuric acid) and dried at 65°C for 48 h to a constant weight. The dried sample was smashed to pass through a 1-mm sieve (Aizela Electric Appliance Co. Ltd., Ningbo, Zhejiang, China) for later analysis.

The milk yield was recorded daily, and the milk samples (50 mL) were collected three times throughout the day and mixed in the proportion of morning, middle, and evening = 4:3:3 on d 60. The composition (protein, fat, lactose, urea nitrogen, and somatic cell count [SCC]) of milk samples were analyzed immediately by an automatic multifunctional dairy analyzer (Botong Ruihua Scientific Instrument Co. Ltd., Beijing, China). Blood was sampled from all cows before morning feeding on d 1 and 60. Using evacuated tubes containing no anticoagulant, blood samples were taken from the caudal vein and then centrifuged at 3,500×g for 15 min at 4°C to harvest serum. Serum samples were collected in 1.5 mL microtubes and stored at −20°C until analysis.

Ruminal fluid samples were collected by a flexible esophageal tube (Anscitech Co. Ltd., Wuhan, Hubei, China) at 4 h after the morning feeding on d 60. Ruminal fluid was strained through 4 layers of cheesecloth, and rumen pH was measured immediately with a portable pH meter (PH200, Ruizhen Electronic Technology Co., Ltd., Shanghai, China). After pH measurement, ten milliliters of strained ruminal fluid were transferred into sterile tubes containing 1 mL of 25% metaphosphoric acid, and this mixture was vigorously hand-shaken and stored at −20°C for later analysis.

### Chemical analysis and calculations

DM, EE, organic matter (OM), and CP of diets, orts, and feces were analyzed according to the AOAC [14]. The neutral detergent fiber (NDF) and acid detergent fiber (ADF) contents were analyzed according to Van Soest et al [15]. The apparent total tract digestibility (D, %) of dietary nutrient was measured using the acid-insoluble ash (AIA) ratio technique. The AIA in the feces (Af, %) and diets (Ad, %) were analyzed using the method described by Van Keulen and Young [16]. With the content of a nutrient in feces (Nf, %) and diet (Nd, %), the nutrient apparent digestibility was determined using an equation as follows: D = [1−(Ad×Nf)/(Af×Nd)]×100.

Serum samples were analyzed for total protein (TP), albumin (ALB), total cholesterol (TC), triglycerides (TG), urea, glucose (GLU), glutamic pyruvic transaminase (ALT), glutamic oxalacetic transaminase (AST), immunoglobulin A (IgA), G (IgG), and M (IgM) using commercial kits (Jiancheng Bioengineering Institute, Nanjing, Jiangsu, China). Frozen rumen fluid samples were thawed and then centrifuged at 15,000×g for 10 min at 4°C, and the supernatant was analyzed for volatile fatty acid (VFA) [17], microbial protein (MCP) [18], and ammonia-N concentrations [19].

### Statistical analysis

The data for analysis were used general linear model procedure of the SPSS statistical software (version 20.0 for Windows; SPSS, Chicago, IL, USA). The model for the statistical analysis is as follows: Y_i_ = μ+T_i_+e_i_, where Y = dependent variable, μ = general mean, T = treatment effect, and e = residual error. Polynomial contrasts and the linearity of the response to analyzed dietary CMS levels were examined. Data were presented as mean and standard error of the mean. The significance level was indicated at p-value <0.05.

## RESULTS

### Feed intake, milk yield, and milk composition

**Figure 1:**
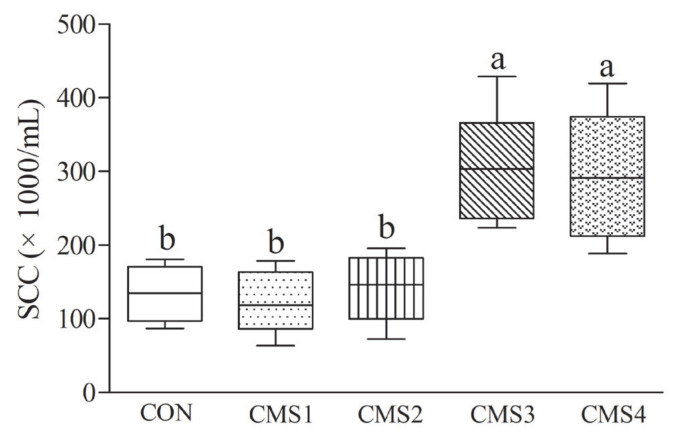
Effects of condensed molasses fermentation solubles on the somatic cell count in milk of dairy cows. CMS, condensed molasses fermentation solubles; SCC, somatic cell count. ^a,b^ Different letters indicate significant differences (p<0.05).

The DMI of CMS2 was maximum and higher (p<0.05) than CMS4 (Table 2). Consistent with DMI, the milk yield and protein of CMS2 group were maximum, and they were higher (p<0.05) than CMS3 and CMS4 groups. However, the milk fat and lactose were similar. Compared to other groups, the milk urea nitrogen in CMS3 and CMS4 groups exhibited higher (p<0.05) concentrations. The SCC of CMS3 and CMS4 groups was more than 200×10^3^/mL, and higher (p<0.05) than other groups (Figure 1).

### Rumen fermentation

The ruminal pH of CMS3 and CMS4 was less than 6, and they had obvious differences (p<0.05) with CON and CMS2 (Table 3). The contents of ammonia N in CMS3 and CMS4 groups were maximum, and they were higher (p<0.05) than other groups, while the MCP concentrations showed an opposite trend. No statistical difference was found in Total VFA. However, the acetate, propionate, and butyrate exhibited significant difference, among which the acetate contents of CMS3 and CMS4 groups were lower (p<0.05) than other groups. Besides, a similar trend was found for the acetate-to-propionate ratio.

### Nutrient apparent digestibility

The apparent digestibility of EE, NDF, and ADF were not different among all the groups (Table 4). However, cows fed different levels of CMS showed numerical differences. The apparent digestibility of DM, OM, and CP of CMS2 were higher (p<0.05) than CMS3 and CMS4. Besides, although there were no obvious difference between CMS2 and CMS1 groups, the numerical value of CMS2 group was greater.

### Serum parameters

**Figure 2:**
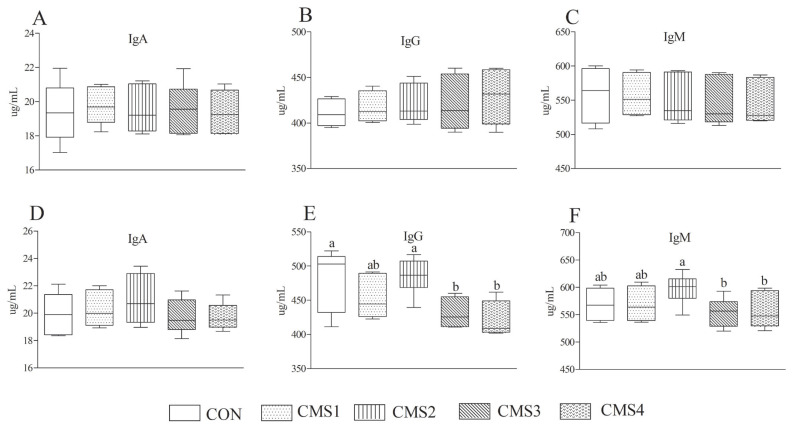
Effects of condensed molasses fermentation solubles on the serum immune parameters of dairy cows. The serum concentrations of IgA, IgG, and IgM on d 1 are shown in (A), (B), and (C), respectively. (D), (E), and (F) represent the concentrations of IgA, IgG, and IgM on d 60, respectively. IgA, immunoglobulin A; IgG, immunoglobulin G; IgM, immunoglobulin G. ^a,b^ Different letters indicate significant differences (p<0.05).

CMS supplementation had no significant effects on serum concentrations of TP, ALB, TC, TG, GLU, ALT, and AST (Table 5). The serum concentration of urea had no noticeable difference on d 1, however, the urea contents of CMS3 and CMS4 groups were lower (p<0.05) than other groups on d 60. The serum concentrations of IgA, IgG, and IgM are shown in Figure 2. Compared to CMS3 and CMS4 groups, the CMS2 group increased (p<0.05) the serum concentrations of IgG and IgM on d 60 (Figure 2).

## DISCUSSION

### Feed intake, milk yield, and milk composition

Feed intake is very important for dairy cows to maintain health and production performance. Previous study found that with the increase of condensed molasses solubles, the DMI of Holstein male calves was not affected by the treatments [2]. Inconsistent with that study, in the present study, we found that excess use of CMS reduced the DMI. The reason may be that the amount of CMS was different. Martel et al [20] pointed out that proper quantity of molasses in diet did not affect DMI of lactating cows, which was in line with our finding. In addition, we also found that cows fed a higher level of CMS had a significantly decreased milk yield and protein. These results suggested that excessive CMS supply might limit milk protein synthesis. An appropriate level of molasses could maintain the performance of dairy cows, which was in accordance with a previous study that used molasses as a substitute for roughage in ration of dairy cows [21]. Milk urea nitrogen was linearly increased as the level of CMS increased, which was consistent with the study of Baurhoo and Mustafa [22]. This means that the excess CMS reduced the protein utilization, which was consistent with a previous study [9]. For SCC, the CMS3 and CMS4 were more than 200×10^3^/mL, suggesting that cows might have inflammation in their mammary glands. Therefore, in the present study, higher addition of CMS (more than 3% of the diet) could decrease production performance of dairy cows.

### Rumen fermentation

The diet affects pH of the rumen, which normally ranges from 6.0 to 7.0. In this study, rumen pH values of CMS3 and CMS4 were less than 6, indicating that overmuch CMS could adversely affect the rumen fermentation. No noticeable effect was observed for ammonia-N among CON, CMS1, and CMS2 groups; however, these groups were lower than CMS3 and CMS4 groups, suggesting that excess CMS might reduce the utilization of ruminal ammonia-N. Ammonia-N is the main raw material for synthesizing MCP. Interestingly, the MCP contents of CON, CMS1, and CMS2 groups were higher than CMS3 and CMS4 groups, which matched ammonia-N results. A study conducted *in vitro* pointed out that molasses in combination with roughage could increase the efficiency of MCP synthesis [23]. In the future, more studies should focus on the combination of CMS and roughage. VFA is the final fermentation product of carbohydrates, and the concentrations of acetate and propionate can reflect rumen fermentation pattern. Lettat and Benchaar [24] reported that higher content of carbohydrate could improve the activity of starch decomposition bacteria and then promote the synthesis of propionate and butyrate, which was in line with our finding. In order to maintain the rumen health, in our study, the best level of CMS in the diet of dairy cows was 2%.

### Nutrient apparent digestibility

Higher nutrient digestibility is beneficial for animal production [13]. Previous studies reported that after microbial fermentation, feed could produce the bacteria protein, small peptides, and amino acids, which were easy to absorb by animals to increase nutrient digestibility [25]. However, in the present study, cows supplemented with higher addition of CMS (CMS3 and CMS4 groups) had reduced DM, OM, and CP digestibility. The results of our study indicated that the appropriate level of CMS in the diet would not adversely affect nutrient digestibility. Generally, the increase of nutrient digestibility can lead to the corresponding increase of milk production [1], which was basically in accordance with the results of this experiment. Stemme et al [9] suggested that because of high content of potassium that could lead to diarrhea, a low proportion of condensed molasses solubles should be used in the animals’ diet. This may explain why a high concentration of CMS causes a decrease in digestibility. But further research is needed to investigate how to improve the nutrient digestibility of CMS. Different combination of feed with CMS may increase nutrient digestibility.

### Serum parameters

Serum biochemical parameters can be used to monitor the health of animals [26]. Little information is available on the effects of CMS on serum biochemical parameters of lactating cows. The contents of TP, ALB, urea, and GLU in serum are important parameters of protein and energy metabolism. The TG and TC can be used to reflect lipid metabolism [26]. Besides, AST and ALT are important enzymes of transaminase, which have essential impact on liver function, and they are the indicators of liver function and related to protein metabolism [27]. In the present study, no obvious differences of TP, ALB, GLU, AST, and ALT were found in all groups except for urea. Therefore, CMS had no effects on the hepatic metabolism of dairy cows. Excessive CMS reduced the serum urea concentrations, and this result was in accordance with the finding of Moloney et al [28]. But the specific molecular mechanism is unclear. In addition, a previous study reported that an appropriate level of fulvic acid was one ingredient of CMS that can improve immunity of animals [29]. In our study, higher levels of CMS reduced serum immune parameters of dairy cows, and these results were consistent with the milk production. The higher level of CMS in diet reduced the immunity of dairy cows, which then led to the decrease of lactation performance. Therefore, suitable addition amount of CMS in dairy cow’ ration was very important.

## CONCLUSION

The partial replacement of concentrates with CMS did not adversely affect DMI, milk yield or composition in lactating cows. Additionally, there was no significant difference in rumen fermentation, nutrient digestibility, and serum parameters between CMS2 and CON groups. Based on our findings, it is practicable that CMS substitutes for a part of concentrates in lactating cows’ diets. However, the amount of CMS should be considered, and a higher addition of CMS (more than 3% of the diet) could decrease production performance of dairy cows in the present study.

## Figures and Tables

**Table 1:** Ingredients and nutrient composition of the basal diet (DM basis)

Items	Group1)				

	CON	CMS1	CMS2	CMS3	CMS4
Ingredients (%)					
Corn silage	35.80	35.80	35.80	35.80	35.80
Alfalfa hay	8.33	8.33	8.33	8.33	8.33
Oat hay	3.83	3.83	3.83	3.83	3.83
Corn	16.67	16.67	16.67	16.67	16.67
Soybean meal	11.67	11.25	10.83	10.42	10.00
Beet pulp	6.36	5.95	5.53	5.11	4.70
Cottonseed	4.17	4.17	4.17	4.17	4.17
DDGS	6.25	6.08	5.92	5.75	5.58
Wheat bran	4.44	4.44	4.44	4.44	4.44
Limestone	1.70	1.70	1.70	1.70	1.70
NaHCO_3_	0.38	0.38	0.38	0.38	0.38
CMS	0	1.00	2.00	3.00	4.00
Premix2)	0.40	0.40	0.40	0.40	0.40
Total	100	100	100	100	100
Nutrient levels (%)					
NE_L_ (MJ/kg)3)	6.93	6.93	6.93	6.92	6.92
CP	16.85	16.85	16.85	16.85	16.84
NFC	39.75	39.98	40.20	40.42	40.65
NDF	34.70	34.41	34.12	33.83	33.54
ADF	23.16	22.97	22.79	22.67	22.43
Ca	0.97	0.98	0.98	0.98	0.98
P	0.44	0.44	0.43	0.43	0.42

DM, dry matter; CMS, condensed molasses fermentation solubles; DDGS, distillers dried grains with soluble; NE_L_, net energy for lactation; CP, crude protein; NFC, non-fibrous carbohydrate; NDF, neutral detergent fiber; ADF, acid detergent fiber.

1) CON, control group, no CMS; CMS1, accounted for 1% of the basal diet; CMS2, 2%; CMS3, 3%; CMS4, 4%.

2) The premix provided the following per kg of the diet: vit A 8,000 IU, vit D 1,200 IU, vit E 50 IU, Cu (as copper sulfate) 10 mg, Fe (as ferrous sulfate) 100 mg, Mn (as manganese sulfate) 40 mg, Zn (as zinc sulfate) 60 mg, I (as potassium iodide) 0.50 mg, Se (as sodium selenite) 0.3 mg, Co (as cobalt chloride) 0.1 mg.

3) NE_L_ was calculated according to the Nutrient Requirements of Dairy Cattle: Seventh Revised Edition, 2001.

**Table 2:** Effects of condensed molasses fermentation solubles on the dry matter intake, milk yield, and composition of dairy cows

Item	Groups1)					SEM	p-value	
	
	CON	CMS1	CMS2	CMS3	CMS4		Linear	Quadratic
Dry matter intake (kg/d)	21.09^a^	21.42^a^	21.76^a^	20.74^ab^	19.65^b^	0.20	0.102	0.008
Milk yield (kg/d)	36.74^a^	36.90^a^	37.05^a^	35.36^b^	32.87^c^	0.21	0.085	<0.001
Milk protein (%)	3.15^a^	3.25^a^	3.28^a^	2.89^b^	2.92^b^	0.08	0.797	0.001
Milk fat (%)	4.22	4.18	4.25	4.24	4.09	0.11	0.806	0.384
Lactose (%)	5.00	5.02	4.97	0.89	0.95	0.04	0.525	0.629
Urea nitrogen (mg/dL)	13.83^c^	10.46^d^	12.59^c^	16.77^b^	19.33^a^	0.18	0.007	0.013

CMS, condensed molasses fermentation solubles; SEM, standard error of the mean.

1) CON, control group, no CMS; CMS1, accounted for 1% of the basal diet; CMS2, 2%; CMS3, 3%; CMS4, 4%.

a–c In the same row, values with different letter mean significant difference (p<0.05).

**Table 3:** Effects of condensed molasses fermentation solubles on the rumen fermentation of dairy cows

Item	Groups1)					SEM	p-value	
	
	CON	CMS1	CMS2	CMS3	CMS4		Linear	Quadratic
pH	6.23^a^	6.02^ab^	6.27^a^	5.86^b^	5.91^b^	0.05	0.040	0.157
Ammonia N (mg/dL)	15.45^b^	16.80^b^	14.27^b^	21.34^a^	19.67^a^	0.41	0.027	0.161
MCP (mg/mL)	8.60^a^	8.41^a^	8.73^a^	6.05^b^	6.27^b^	0.25	0.003	0.038
Total VFA (mmol/L)	104.27	106.51	107.34	106.70	105.38	1.62	0.383	0.648
Acetate (mmol/L)	65.72^a^	66.19^a^	68.69^a^	59.30^b^	58.37^b^	0.70	0.036	<0.001
Propionate (mmol/L)	19.86^b^	23.76^ab^	20.75^b^	26.09^a^	27.67^a^	0.55	0.011	0.348
Butyrate (mmol/L)	12.08^b^	11.21^b^	13.01^b^	16.17^a^	15.50^a^	0.24	0.001	0.070
Acetate-to-propionate ratio	3.31^a^	2.78^a^	3.30^a^	2.27^b^	2.11^b^	0.04	<0.001	0.033

CMS, condensed molasses fermentation solubles; SEM, standard error of the mean; MCP, microbial protein; VFA, volatile fatty acid.

1) CON, control group, no CMS; CMS1, accounted for 1% of the basal diet; CMS2, 2%; CMS3, 3%; CMS4, 4%.

a,b In the same row, values with different letter mean significant difference (p<0.05).

**Table 4:** Effects of condensed molasses fermentation solubles on the nutrient apparent digestibility of dairy cows

Item	Groups1)					SEM	p-value	
	
	CON	CMS1	CMS2	CMS3	CMS4		Linear	Quadratic
DM (%)	74.23^a^	72.49^a^	75.05^a^	66.11^b^	62.18^b^	1.03	0.021	0.037
OM (%)	63.76^ab^	63.92^ab^	65.37^a^	58.08^b^	60.72^b^	1.31	0.103	0.040
CP (%)	67.85^b^	71.27^ab^	73.03^a^	63.18^c^	61.73^c^	1.46	0.239	0.004
EE (%)	71.33	68.27	70.20	68.92	68.37	1.60	0.587	0.690
NDF (%)	53.26	50.59	52.17	50.76	51.22	1.36	0.867	0.668
ADF (%)	47.33	44.54	48.66	45.29	43.08	1.65	0.118	0.243

CMS, condensed molasses fermentation solubles; SEM, standard error of the mean; DM, dry matter; OM, organic matter; CP, crude protein; EE, ether extract; NDF, neutral detergent fiber; ADF, acid detergent fiber.

1) CON, control group, no CMS; CMS1, accounted for 1% of the basal diet; CMS2, 2%; CMS3, 3%; CMS4, 4%.

a–c In the same row, values with different letter mean significant difference (p<0.05).

**Table 5:** Effects of condensed molasses fermentation solubles on the serum biochemical parameters of dairy cows

Item	Time	Groups1)					SEM	p-value	
	
		CON	CMS1	CMS2	CMS3	CMS4		Linear	Quadratic
TP (g/L)	d 1	75.93	71.26	74.96	73.28	71.94	1.13	0.876	0.244
	d 60	73.48	72.37	71.09	70.97	72.60	1.42	0.671	0.510
ALB (g/L)	d 1	29.13	29.32	29.14	28.49	30.02	0.35	0.501	0.486
	d 60	28.37	30.22	31.02	29.08	28.34	1.02	0.707	0.411
TC (mmol/L)	d 1	6.03	6.21	6.18	5.86	6.26	0.21	0.189	0.640
	d 60	5.99	6.19	6.20	6.22	6.18	0.29	0.381	0.492
TG (mmol/L)	d 1	0.13	0.14	0.13	0.15	0.14	0.02	0.833	0.741
	d 60	0.14	0.14	0.15	0.14	0.13	0.01	0.857	0.839
Urea (mmol/L)	d 1	3.30	3.57	3.40	3.29	3.38	0.06	0.501	0.633
	d 60	3.41^a^	3.39^a^	3.43^a^	2.86^b^	2.75^b^	0.03	0.037	0.019
GLU (mmol/L)	d 1	3.56	3.61	3.52	3.62	3.59	0.03	0.930	0.711
	d 60	3.62	3.57	3.66	3.71	3.82	0.08	0.215	0.336
ALT (U/L)	d 1	30.06	29.43	31.89	31.77	32.06	1.06	0.156	0.761
	d 60	28.76	27.31	28.06	30.18	31.47	1.02	0.620	0.124
AST (U/L)	d 1	79.42	81.14	81.33	81.57	79.06	2.64	0.201	0.188
	d 60	80.09	79.37	78.37	83.67	81.29	2.49	0.338	0.140

CMS, condensed molasses fermentation solubles; SEM, standard error of the mean; TP, total protein; ALB, albumin; TC, total cholesterol; TG, triglycerides; GLU, glucose; ALT, glutamic pyruvic transaminase; AST, glutamic oxalacetic transaminase.

1) CON, control group, no CMS; CMS1, accounted for 1% of the basal diet; CMS2, 2%; CMS3, 3%; CMS4, 4%.

a,b In the same row, values with different letter mean significant difference (p<0.05).
